# Supplementary material for: Safety Assessment of 3S, 3’S Astaxanthin Derived from Metabolically Engineered K. marxianus
Source: Antioxidants (Basel). 2022 Nov 18;11(11):2288. doi: 10.3390/antiox11112288 (PMC9687027; doi:10.3390/antiox11112288)
Supplement: Supplementary file 1 [file antioxidants-11-02288-s001.zip › antioxidants-2004227-supplementary.pdf]

Supplementary Table

Table S1.
Basic design for animal study

**Group No.**	**Dose (mg/kg/day)**	**Dose Conc. (mg/mL)**	**Dose Volume (mL/kg)**	**No. of Animals**
1 (Vehicle control)	0	0	3	10M, 10F
2 (Low-dose)	6	2	3	10M, 10F
3 (Mid-dose)	12	4	3	10M, 10F
4 (High-dose)	24	8	3	10M, 10F

Table S2.
Items analyzed for Toxicity Study

	Red blood cell counts (RBC)
	White blood cell counts (WBC)
	Platelet counts (PLT)
	Hemoglobin (HGB)
	Hematocrit (HCT)
	Mean Corpuscular Volume (MCV)
	Mean Corpuscular Hemoglobin (MCH)
	Mean Corpuscular Hemoglobin Concentration (MCHC) Reticulocyte (RET)
Hematology	WBC differential
	neutrophils (NEUT)
	eosinophils (EOSIN)
	basophils (BASO)
	monocytes (MONO)
	lymphocytes (LYMPH)
	Activated partial thromboplastin time (APTT)
Coagulation	Prothrombin time (PT)
	Fibrinogen (FIB)
	Amylase (AMY)
	Albumin (ALB)
	Alkaline phosphatase (ALP)
	Total bilirubin (T-BIL)

Serum Chemistry	Alanine aminotransferase (ALT)<br>Aspartate aminotransferase (AST)<br>Gamma-glutamyl transferase (GGT)<br>Total bile acids (TBA)<br>Total protein (TP)<br>Creatinine (CRE)<br>Blood Urea nitrogen (BUN)<br>Total Cholesterol (CHO)<br>Triglycerides (TG)<br>Creatine kinase (CK)<br>Lactate dehydrogenase (LDH)<br>Chloride (Cl)<br>Sodium (Na)<br>Potassium (K)<br>Glucose (GLU)<br>Calcium (Ca)<br>Phosphorus (P)
Urinalysis	pH<br>Specific gravity<br>Protein<br>Glucose<br>Ketone<br>Bilirubin<br>Urobilinogen<br>Occulted blood<br>Leukocytes<br>Nitrite
Urinary Sediments Examination	Red blood cell<br>White blood cell<br>Cells<br>Casts<br>Crystals<br>Microbes

Table S3.

A. Mean body weight of rats receiving astaxanthin over 13-week period

Mean Body Weights (g, Mean ± SD)	
**Male**	**Female**

Study Day	Vehicle Control 0 mg/kg/day	Low-dose 6 mg/kg/day	Mid-dose 12 mg/kg/day	High-dose 24 mg/kg/day	Vehicle Control 0 mg/kg/day	Low-dose 6 mg/kg/day	Mid-dose 12 mg/kg/day	High-dose 24 mg/kg/day
**Number of animals**	10	10	10	10	10	10	10	10
**Day 1**	182.25 ± 7.90	183.03 ± 9.41	181.76 ± 8.31	182.49 ± 9.05	156.73 ± 7.46	153.36 ± 5.70	156.79 ± 6.96	154.93 ± 7.47
**Day 8**	241.90 ± 10.10	242.92 ± 15.85	238.14 ± 8.88	236.88 ± 8.53	178.69 ± 8.24	175.25 ± 9.96	173.92 ± 11.31	176.21 ± 7.00
**Day 15**	299.41 ± 13.36	298.53 ± 23.42	291.20 ± 16.54	289.90 ± 14.95	192.81 ± 7.63	190.58 ± 12.85	187.03 ± 15.17	193.74 ± 5.35
**Day 22**	351.21 ± 18.00	347.76 ± 33.52	340.45 ± 25.02	337.03 ± 22.26	206.16 ± 11.32	204.25 ± 11.23	202.71 ± 16.74	205.51 ± 4.91
**Day 29**	392.90 ± 22.96	390.61 ± 43.53	381.23 ± 32.01	376.63 ± 27.66	215.43 ± 13.07	215.26 ± 11.86	215.51 ± 17.32	219.18 ± 7.41
**Day 36**	429.75 ± 27.87	422.16 ± 49.88	411.88 ± 36.96	406.39 ± 32.03	230.87 ± 14.37	227.68 ± 11.89	226.26 ± 19.57	228.64 ± 7.44
**Day 43**	458.07 ± 32.25	450.94 ± 52.13	437.34 ± 42.09	431.71 ± 33.91	237.20 ± 16.45	237.29 ± 12.44	233.95 ± 18.43	241.09 ± 6.78
**Day 50**	485.62 ± 33.43	474.10 ± 58.69	457.29 ± 46.49	453.31 ± 39.23	243.31 ± 18.40	241.73 ± 12.90	240.75 ± 19.40	243.02 ± 6.38
**Day 57**	508.60 ± 35.79	501.29 ± 64.23	477.82 ± 48.82	474.56 ± 41.96	248.55 ± 18.13	246.12 ± 13.47	245.19 ± 19.19	246.87 ± 8.65
**Day 64**	526.29 ± 37.66	515.44 ± 68.02	492.98 ± 52.61	491.19 ± 44.31	257.46 ± 18.14	252.23 ± 14.43	250.16 ± 20.11	254.14 ± 8.54
**Day 71**	544.66 ± 41.26	532.41 ± 69.48	507.16 ± 57.15	505.40 ± 42.48	258.77 ± 19.63	256.98 ± 13.08	253.92 ± 17.91	257.81 ± 8.87
**Day 78**	558.39 ± 40.71	546.69 ± 70.60	519.89 ± 60.31	520.76 ± 47.54	261.86 ± 20.38	258.85 ± 15.95	258.05 ± 19.73	259.67 ± 8.99
**Day 85**	573.36 ± 44.74	559.04 ± 74.11	531.01 ± 61.23	534.14 ± 48.61	266.93 ± 20.97	263.25 ± 16.15	262.08 ± 20.86	262.35 ± 10.30
**Day 91**	583.10 ± 45.14	596.31 ± 78.13	540.59 ± 60.80	543.34 ± 51.12	267.03 ± 21.13	265.87 ± 14.67	261.79 ± 19.43	264.88 ± 8.43

B. Mean body weight gain of rats receiving astaxanthin over 13-week period

Study Day	Mean Body Weights (g, Mean ± SD)							
	Male				Female			
	Vehicle Control 0 mg/kg/day	Low-dose 6 mg/kg/day	Mid-dose 12 mg/kg/day	High-dose 24 mg/kg/day	Vehicle Control 0 mg/kg/day	Low-dose 6 mg/kg/day	Mid-dose 12 mg/kg/day	High-dose 24 mg/kg/day
Number of animals	10	10	10	10	10	10	10	10
**Day 1-8**	59.65 ± 6.48	59.89 ± 11.98	56.38 ± 7.43	54.39 ± 6.30	21.96 ± 5.25	21.89 ± 6.89	17.13 ± 7.36	21.28 ± 3.99
**Day 8-15**	57.51 ± 8.02	55.61 ± 10.33	53.06 ± 8.60	53.02 ± 7.90	14.12 ± 5.14	15.33 ± 5.38	13.11 ± 6.73	17.53 ± 3.74
**Day 15-22**	51.80 ± 6.23	49.23 ± 11.53	49.25 ± 10.25	47.13 ± 8.68	13.35 ± 5.66	13.67 ± 4.35	15.68 ± 4.08	11.77 ± 5.55
**Day 22-29**	41.69 ± 5.98	42.85 ± 12.21	40.78 ± 10.84	39.60 ± 7.37	9.27 ± 4.54	11.01 ± 4.81	12.80 ± 3.24	13.67 ± 6.55
**Day 29-36**	36.85 ± 6.76	31.55 ± 7.05	30.65 ± 6.43	29.76 ± 6.76	15.44 ± 4.80	12.42 ± 5.40	10.75 ± 3.99	9.46 ± 7.04
**Day 36-43**	28.32 ± 5.00	28.78 ± 3.93	25.46 ± 6.23	25.32 ± 4.35	6.33 ± 3.02	9.61 ± 3.23	7.69 ± 4.25	12.45 ± 4.63*
**Day 43-50**	27.55 ± 4.25	23.16 ± 7.20	19.95 ± 6.02	21.60 ± 6.78	6.11 ± 5.67	4.44 ± 4.78	6.80 ± 5.19	1.93 ± 4.53
**Day 50-57**	22.98 ± 5.41	27.19 ± 6.13	20.53 ± 3.70	21.25 ± 4.22	5.24 ± 5.64	4.39 ± 5.77	4.44 ± 2.76	3.85 ± 4.47
**Day 57-64**	17.69 ± 7.01	14.15 ± 6.46	15.16 ± 5.05	16.63 ± 5.52	8.91 ± 4.27	6.11 ± 5.55	4.97 ± 4.02	7.27 ± 5.53
**Day 64-71**	18.37 ± 5.62	16.97 ± 4.82	14.18 ± 5.77	14.21 ± 4.55	1.31 ± 4.97	4.75 ± 2.68	3.76 ± 5.62	3.67 ± 4.57
**Day 71-78**	13.73 ± 3.26	14.28 ± 4.67	12.73 ± 5.88	15.36 ± 6.87	3.09 ± 5.56	1.87 ± 6.71	4.13 ± 4.61	1.86 ± 2.55
**Day 78-85**	14.97 ± 5.47	12.35 ± 6.13	11.12 ± 4.42	13.38 ± 4.03	5.07 ± 5.35	4.40 ± 4.93	4.03 ± 4.48	2.68 ± 3.73
**Day 85-91**	9.74 ± 3.74	10.27 ± 6.94	9.58 ± 3.06	9.20 ± 5.64	0.10 ± 4.65	2.62 ± 6.67	-0.29 ± 4.49	2.53 ± 3.73
**Day 1-91**	400.85 ± 44.38	386.28 ± 77.95	358.63 ± 61.13	360.85 ± 53.01	110.30 ± 19.14	112.51 ± 15.20	105.00 ± 17.95	109.95 ± 9.17

*: $p \leq 0.05$ (compared to vehicle control group)

Table S4.

Mean food consumption of rats receiving astaxanthin over 13-week period

Study Day	Mean Food Consumption (g/day, Mean ± SD)							
	Male				Female			
	Vehicle Control 0 mg/kg/day	Low-dose 6 mg/kg/day	Mid-dose 12 mg/kg/day	High-dose 24 mg/kg/day	Vehicle Control 0 mg/kg/day	Low-dose 6 mg/kg/day	Mid-dose 12 mg/kg/day	High-dose 24 mg/kg/day
**Number of cages**	5	5	5	5	5	5	5	5
**Day 1-8**	24.88 ± 1.64	24.62 ± 1.03	23.01 ± 0.82	23.27 ± 0.90	15.71 ± 0.66	16.51 ± 0.88	15.78 ± 0.84	15.90 ± 0.76
**Day 8-15**	27.98 ± 1.50	27.86 ± 1.49	25.76 ± 1.34	26.33 ± 1.05	16.22 ± 0.68	16.72 ± 0.85	16.39 ± 0.92	16.24 ± 0.64
**Day 15-22**	29.82 ± 1.84	29.77 ± 1.82	27.21 ± 1.98	27.35 ± 1.55	16.31 ± 0.60	17.38 ± 1.53	16.65 ± 0.83	16.32 ± 0.55
**Day 22-29**	29.36 ± 1.17	29.56 ± 2.42	26.56 ± 2.13	27.20 ± 1.91	16.79 ± 0.62	17.98 ± 0.95	17.00 ± 0.79	16.68 ± 0.84
**Day 29-36**	28.97 ± 1.49	28.83 ± 2.39	25.74 ± 1.65	26.75 ± 2.00	17.20 ± 0.43	17.61 ± 1.24	17.01 ± 0.72	16.51 ± 0.71
**Day 36-43**	29.26 ± 2.11	29.13 ± 2.37	26.11 ± 1.67	26.89 ± 1.74	16.65 ± 0.53	17.42 ± 1.23	16.73 ± 0.63	16.97 ± 1.11
**Day 43-50**	29.56 ± 2.59	29.34 ± 2.49	25.50 ± 2.14	25.79 ± 2.02	16.96 ± 1.02	17.55 ± 1.66	16.90 ± 0.85	16.20 ± 0.70
**Day 50-57**	28.69 ± 1.64	28.64 ± 1.75	25.36 ± 1.95*	26.29 ± 1.91	16.62 ± 0.69	16.80 ± 0.90	16.15 ± 0.58	15.89 ± 1.01
**Day 57-64**	27.23 ± 2.13	26.99 ± 2.04	24.33 ± 1.57	25.22 ± 1.82	16.15 ± 0.61	16.50 ± 0.78	15.78 ± 0.77	15.37 ± 1.21
**Day 64-71**	26.63 ± 2.41	26.81 ± 3.04	23.38 ± 1.96	24.80 ± 1.77	15.80 ± 0.95	16.39 ± 0.92	15.83 ± 0.85	15.00 ± 1.62
**Day 71-78**	26.43 ± 2.40	26.55 ± 2.09	23.96 ± 2.10	25.39 ± 1.79	15.85 ± 1.09	15.71 ± 1.46	15.41 ± 1.06	15.07 ± 1.27
**Day 78-85**	26.09 ± 2.83	25.49 ± 1.96	22.68 ± 1.63	24.34 ± 2.12	15.98 ± 0.68	15.92 ± 1.15	15.78 ± 0.83	14.95 ± 1.00
**Day 85-91**	27.63 ± 3.09	27.41 ± 2.68	24.06 ± 1.70	25.80 ± 1.89	15.92 ± 0.55	16.10 ± 1.10	15.51 ± 0.82	15.68 ± 1.46

*: $p \leq 0.05$ (compared to vehicle control group)

Table S5.

Ophthalmologic examinations of rats receiving astaxanthin over 13-week period

Gender	Dose (mg/kg/day)	Ophthalmologic Examination (N/N)[1]	
		Before study	Before necropsy (Day 91)
Male	0	0/10	0/10
	6	0/10	0/10
	12	0/10	0/10
	24	0/10	0/10
Female	0	0/10	0/10
	6	0/10	0/10
	12	0/10	0/10
	24	0/10	0/10

[1] N/N: Number of animals with ophthalmologic abnormality/Total number of animals

Table S6.

A. Absolute organ weight of rats receiving astaxanthin over 13-week period

Parameters	Organs Weights (gram, Mean ± SD)							
Group	Vehicle Control 0 mg/kg/day	Low-dose 6 mg/kg/day	Mid-dose 12 mg/kg/day	High-dose 24 mg/kg/day	Vehicle Control 0 mg/kg/day	Low-dose 6 mg/kg/day	Mid-dose 12 mg/kg/day	High-dose 24 mg/kg/day
Gender	Male				Female			
Number of animals	10	10	10	10	10	10	10	10
Adrenals	0.04955 ± 0.00546	0.05224 ± 0.00982	0.05446 ± 0.00564	0.05437 ± 0.00810	0.06268 ± 0.00862	0.05650 ± 0.00702	0.06074 ± 0.00816	0.06012 ± 0.00631
Pituitary	0.01462 ± 0.00304	0.01265 ± 0.00325	0.01368 ± 0.00192	0.01479 ± 0.00070	0.01555 ± 0.00223	0.01525 ± 0.00240	0.01534 ± 0.00231	0.01581 ± 0.00182
Brain	2.239 ± 0.107	2.132 ± 0.139	2.235 ± 0.059	2.209 ± 0.147	2.014 ± 0.071	1.971 ± 0.052	1.985 ± 0.107	2.025 ± 0.062
Heart	1.609 ± 0.181	1.632 ± 0.203	1.550 ± 0.170	1.523 ± 0.154	0.912 ± 0.084	0.884 ± 0.062	0.872 ± 0.090	0.895 ± 0.082
Thymus	0.311 ± 0.060	0.289 ± 0.119	0.350 ± 0.063	0.316 ± 0.084	0.211 ± 0.066	0.241 ± 0.043	0.232 ± 0.067	0.228 ± 0.050

Liver	16.431 ± 2.811	14.985 ± 3.182	13.959 ± 2.310	13.951 ± 2.038	6.813 ± 0.477	7.428 ± 1.029	7.441 ± 1.160	7.147 ± 0.484
Spleen	0.860 ± 0.124	0.775 ± 0.201	0.729 ± 0.113	0.745 ± 0.137	0.470 ± 0.088	0.474 ± 0.040	0.477 ± 0.087	0.494 ± 0.094
Kidneys	3.555 ± 0.336	3.422 ± 0.494	3.316 ± 0.453	3.419 ± 0.370	1.745 ± 0.171	1.815 ± 0.154	1.831 ± 0.158	1.904 ± 0.109
Testes	3.596 ± 0.307	3.448 ± 0.309	3.493 ± 0.205	3.532 ± 0.194	NA	NA	NA	NA
Epididymides	1.321 ± 0.139	1.288 ± 0.077	1.272 ± 0.063	1.288 ± 0.078	NA	NA	NA	NA
Prostate and Seminal	3.471 ± 0.364	3.476 ± 0.379	3.443 ± 0.365	3.595 ± 0.144	NA	NA	NA	NA
Ovary/ Oviduct	NA	NA	NA	NA	0.11430 ± 0.01402	0.12051 ± 0.01612	0.11556 ± 0.02036	0.11830 ± 0.01136
Uterus/ Cervix	NA	NA	NA	NA	0.809 ± 0.359	0.737 ± 0.313	0.583 ± 0.181	0.720 ± 0.252

B. Estrus Cycle of female rats receiving astaxanthin over 13-week period

	Entrus Cycle Stages (Number of rats)			
Group	Proestrus	Estrus	Metestrus	Diestrus
1	7	0	3	0
2	6	1	3	0
3	7	1	2	0
4	7	1	2	0

C. Relative organ weights (organ-to-terminal body weight) of rats receiving astaxanthin over 13-week period

**Parameters**	**Organs Weights/Terminal Body Weight Ratio (%, Mean ± SD)**							
**Group**	**Vehicle Control 0 mg/kg/day**	**Low-dose 6 mg/kg/day**	**Mid-dose 12 mg/kg/day**	**High-dose 24 mg/kg/day**	**Vehicle Control 0 mg/kg/day**	**Low-dose 6 mg/kg/day**	**Mid-dose 12 mg/kg/day**	**High-dose 24 mg/kg/day**
**Gender**	**Male**				**Female**			
**Number of animals**	10	10	10	10	10	10	10	10
Adrenals	0.00888 ± 0.00097	0.00956 ± 0.00127	0.01061 ± 0.00091*	0.01051 ± 0.00163*	0.02486 ± 0.00339	0.02273 ± 0.00284	0.02474 ± 0.00310	0.02422 ± 0.00260
Pituitary	0.00262 ±	0.00230 ±	0.00266 ±	0.00286 ±	0.00615 ±	0.00614 ±	0.00624 ±	0.00637 ±

	0.00052	0.00042	0.00033	0.00023	0.00067	0.00097	0.00093	0.00075
Brain	0.40285 ± 0.03701	0.39495 ± 0.04166	0.43780 ± 0.04396	0.42716 ± 0.03516	0.80089 ± 0.06253	0.79440 ± 0.06009	0.80995 ± 0.06083	0.81632 ± 0.04561
Heart	0.28755 ± 0.01735	0.29980 ± 0.02230	0.30143 ± 0.02158	0.29365 ± 0.02386	0.36170 ± 0.03100	0.35526 ± 0.02071	0.35401 ± 0.01535	0.36036 ± 0.03169
Thymus	0.05601 ± 0.01193	0.05146 ± 0.01675	0.06797 ± 0.00981	0.06036 ± 0.01375	0.08378 ± 0.02619	0.09693 ± 0.01699	0.09395 ± 0.02509	0.09235 ± 0.02307
Liver	2.92675 ± 0.29745	2.71852 ± 0.26417	2.69910 ± 0.22519	2.67791 ± 0.22399	2.69980 ± 0.12228	2.98688 ± 0.40311*	3.01070 ± 0.23581*	2.87849 ± 0.19718
Spleen	0.15367 ± 0.01694	0.13999 ± 0.02206	0.14189 ± 0.01932	0.14293 ± 0.01876	0.18606 ± 0.03330	0.19093 ± 0.02002	0.19329 ± 0.02788	0.19916 ± 0.03911
Kidneys	0.63697 ± 0.04837	0.62867 ± 0.06658	0.64339 ± 0.04905	0.65838 ± 0.04684	0.69069 ± 0.04533	0.73006 ± 0.06278	0.74450 ± 0.03801	0.76700 ± 0.04710*
Testes	0.64700 ± 0.07450	0.63801 ± 0.06988	0.68479 ± 0.08337	0.68349 ± 0.05478	NA	NA	NA	NA
Epididymides	0.23708 ± 0.02562	0.23939 ± 0.03312	0.24985 ± 0.03269	0.24891 ± 0.01691	NA	NA	NA	NA
Prostate and Seminal	0.623 ± 0.065	0.649 ± 0.127	0.675 ± 0.101	0.697 ± 0.067	NA	NA	NA	NA
Ovary/ Oviduct	NA	NA	NA	NA	0.04548 ± 0.00670	0.04855 ± 0.00713	0.04696 ± 0.00741	0.04765 ± 0.00455
Uterus/ Cervix	NA	NA	NA	NA	0.31725 ± 0.13315	0.29473 ± 0.12087	0.23741 ± 0.07090	0.29063 ± 0.10468

*: $p \leq 0.05$ (compared to vehicle control group)

Relative organ weight (%) = Absolute organ weight (g) / Terminal Body weight of rat on the day of sacrifice (g) x 100

D. Relative organ weight (organ-to-brain weight) of rats receiving astaxanthin over 13-week period

**Parameters**	**Organs Weights/Terminal Body Weight Ratio (%, Mean ± SD)**							
**Group**	**Vehicle Control 0 mg/kg/day**	**Low-dose 6 mg/kg/day**	**Mid-dose 12 mg/kg/day**	**High-dose 24 mg/kg/day**	**Vehicle Control 0 mg/kg/day**	**Low-dose 6 mg/kg/day**	**Mid-dose 12 mg/kg/day**	**High-dose 24 mg/kg/day**
**Gender**	**Male**				**Female**			
**Number of animals**	10	10	10	10	10	10	10	10
Adrenals	2.21728 ± 0.26872	2.45088 ± 0.43833	2.43692 ± 0.24853	2.47264 ± 0.41776	3.10798 ± 0.37457	2.86499 ± 0.33150	3.06796 ± 0.45910	2.97251 ± 0.34334
Pituitary	0.65713 ± 0.14793	0.58897 ± 0.13357	0.61248 ± 0.08798	0.67142 ± 0.04202	0.77200 ± 0.10760	0.77528 ± 0.13294	0.77358 ± 0.12024	0.77962 ± 0.07288
Brain	100.00000	100.00000	100.00000 ±	100.00000	100.00000	100.00000	100.00000 ±	100.00000 ±

	± 0.00000	± 0.00000	0.00000	± 0.00000	± 0.00000	± 0.00000	0.00000	0.00000
Heart	71.93438 ± 8.09064	76.40161 ± 6.97738	69.36157 ± 7.47811	69.08830 ± 7.28754	45.27415 ± 3.78880	44.86268 ± 3.11345	43.95798 ± 4.18680	44.29964 ± 5.04055
Thymus	13.87213 ± 2.42212	13.37398 ± 5.08095	15.64278 ± 2.63502	14.28617 ± 3.73081	10.45749 ± 3.13108	12.26994 ± 2.39970	11.64525 ± 3.07804	11.24520 ± 2.32978
Liver	735.53341 ± 131.64079	699.78210 ± 131.27845	624.21273 ± 100.06107	631.59290 ± 81.93341	338.55639 ± 25.14445	376.61486 ± 49.06503	375.04653 ± 54.88274	353.33701 ± 27.64662
Spleen	38.48325 ± 5.75422	36.16696 ± 8.63830	32.65621 ± 5.21805	33.63503 ± 5.08597	23.37912 ± 4.69625	24.04074 ± 1.86449	23.97242 ± 3.87714	24.36295 ± 4.32669
Kidneys	158.97385 ± 15.29231	160.10357 ± 18.04395	148.45323 ± 20.43978	154.85288 ± 14.31550	86.59682 ± 7.46051	92.08376 ± 7.38824	92.21555 ± 5.77266	94.09488 ± 6.04684
Testes	160.81788 ± 14.61765	161.80640 ± 11.57058	156.34507 ± 9.38066	160.31247 ± 10.77010	NA	NA	NA	NA
Epididymides	58.98945 ± 5.50995	60.65638 ± 5.57287	56.95050 ± 3.19883	58.38157 ± 2.73921	NA	NA	NA	NA
Prostate and Seminal	155.361 ± 17.932	163.815 ± 22.269	154.329 ± 18.606	163.525 ± 14.414	NA	NA	NA	NA
Ovary/ Oviduct	NA	NA	NA	NA	5.67960 ± 0.72943	6.11232 ± 0.78629	5.81201 ± 0.86809	5.84633 ± 0.58486
Uterus/ Cervix	NA	NA	NA	NA	40.25734 ± 17.90657	37.68779 ± 16.87806	29.41385 ± 9.18077	35.58113 ± 12.44363

Relative organ weight (%) = Absolute organ weight (g) / Terminal Body weight of rat on the day of sacrifice (g) x 100

Table S7.

Gross necropsy of rats receiving astaxanthin over 13-week period

Male

**Group**	Control	Low-dose	Mid-dose	High-dose
**Gross findings**	Incidence[1]			
**Thymus**				
Discoloration, present, red	**1/10**	0/10	0/10	0/10
**Adrenal glands**				
Discoloration, present, multifocal, white	**1/10**	0/10	0/10	0/10
**Brain**				
Abnormal shape, present, cerebrum, right, depressed	**1/10**	0/10	0/10	0/10

[1] Incidence = number of animals with gross findings / number of animals for gross examinations (N/N).

Female

Group	Control	Low-dose	Mid-dose	High-dose
**Gross findings**	Incidence[1]			
**Mandibular lymph node**				
Discoloration, present, red	**1/10**	0/10	0/10	0/10
**Liver**				
Nodule, present, right lateral lobe	**1/10**	0/10	0/10	0/10

[1] Incidence = number of animals with gross findings / number of animals for gross examinations (N/N).

Table S8.

Histopathology values of rats receiving astaxanthin over 13-week period

Group		Control	Low-dose	Mid-dose	High-dose		Control	Low-dose	Mid-dose	High-dose
**Histopathologic findings**	**S[2]**	Incidence[1]				**S[2]**	Incidence[1]			
**Thymus**										
Congestion	**P**	**1/10**	NA	NA	0/10	**P**	**1/10**	NA	NA	0/10
**Liver**										
Infiltration, mononuclear cell	**1**	**3/10**	NA	NA	**4/10**	**1**	**4/10**	NA	NA	**3/10**
Fatty change, minimal	**1**	**1/10**	NA	NA	0/10	**P**	**1/10**	NA	NA	0/10
**Kidneys**										
Degeneration, tubular epithelial cell	**1**	**1/10**	NA	NA	**1/10**	**1**	0/10	NA	NA	**1/10**
Infiltration, mononuclear cell, interstitial	**1**	**2/10**	NA	NA	**2/10**	**1**	**1/10**	NA	NA	0/10
Mineralization, corticomedullary junction	**1**	0/10	NA	NA	**1/10**					

[1] Incidence = number of animals with histopathologic findings / Total number of animals for histopathologic examinations (N/N).

[2] The severity grading scheme: 1 = minimal (< 10%), 2 = mild (10–39%), 3 = moderate (40–79%), 4 = marked (80–100%), P = present.
